# When open mindedness hinders consensus

**DOI:** 10.1038/s41598-020-64691-0

**Published:** 2020-05-19

**Authors:** Hendrik Schawe, Laura Hernández

**Affiliations:** Laboratoire de Physique Théorique et Modélisation, UMR-8089 CNRS, CY Cergy Paris Université, Paris, France

**Keywords:** Phase transitions and critical phenomena, Complex networks

## Abstract

We perform a detailed study of the Hegselmann-Krause bounded confidence opinion dynamics model with heterogeneousconfidence *ε*_*i*_ drawn from uniform distributions in different intervals [*ε*_*1*_, *ε*_*u*_]. The phase diagram reveals a highly complex andnon-monotonous behaviour, with a re-entrant consensus phase in the region where fragmentation into multiple distinct opinionsis expected for the homogeneous case. A careful exploration of the phase diagram, along with an extensive finite-size analysis,allows us to identify the mechanism leading to this counter-intuitive behaviour. This systematic study over system sizes whichgo well beyond those of previous works, is enabled by an efficient algorithm presented in this article.

## Introduction

Opinion formation and its dissemination within a society, a recurrent subject of study in social sciences, is often addressed through statistical analyses of data collected by the means of surveys or polls. By following the evolution of the statistical outcomes over time, it is possible to obtain some information about the dynamical processes underlying these phenomena. What we call *the opinion of a society* is a global property that characterises the society as a whole and has emerged from the repeated interactions among their agents. Defined in this way, opinion may be studied statistically. Although individual fluctuations are always present, it is very unlikely that they change the stabilized social opinion. This approach is the mathematical realization of the ideas introduced more than one century ago by E. Durkheim^1^, who coined the concept of *social facts*. A social fact is a property characterizing the whole society instead of the individuals, which emerges as an outcome of the dynamics governed by the interactions among them. Once the social fact is created, it is imposed on the members of the society who will find it very difficult to change it.

Early opinion dynamics studies mainly dealt with the case of a fully mixed population, which means that every agent may potentially interact with any other in the population, in other words, the interactions among agents were supposed to be long range. It is well known that this approach is equivalent to what is called in Physics, a mean-field approximation which neglects the structure of the interactions. However, if social opinion emerges from the interactions between the agents, the structure of these interactions may be relevant. In fact, the key role of interactions has been recognized long ago by works that collected data about the detail of social interactions in very small societies, using graph theory to represent them. J. Scott^2^ gives a nice historical overview of network development in social sciences. Nowadays, the development of mathematical models along with the continuous growth of data collection on human activities that proliferated in particular due to the rise of online social networks, allow us to investigate different dynamical processes of opinion dynamics.

The rationale behind most opinion dynamics models is grounded in *Social Influence Theory*^3,4^ which assumes that, as agents interact, they may influence each other making their opinions more alike. Accordingly, the dynamical rules that govern the evolution of the opinion of each agent, are often based on functions which aggregate the opinions of a given set of agents which are assumed to interact with it. Recently a complete mathematical classification of the possible outcomes of the dynamics based on aggregation functions in the case of discrete opinion variables has been obtained^5,6^.

The mathematical description of the structure of social interactions in terms of networks allows to identify the neighbourhood of a given agent (the set of other agents directly connected to it) and it is now clear that this structure is relevant. Different neighbourhood choices along with the type of influence they have on each agent have been considered, for example the adoption of the opinion of a randomly chosen neighbour^7^, the adoption of the neighbours’ majoritarian opinion^8^, or of agents whose opinion on other topics are already near the one of the target agent^9^.

Among all the studied models^10,11^, those representing the agents’ opinion by a continuous variable are well suited to describe situations where the opinion on a particular problem is gradually built through the exchanges among the agents. In particular, *bounded confidence* models consider that each agent will only interact with those agents whose opinions are already close to theirs and will not interact with the others. In the original models a given agent may interact with all the others *provided* that they fall within the confidence interval, meaning that the difference of opinions between potentially interacting agents is lower than a certain threshold that characterizes the society. This simple model is already different from a fully mixed population. Moreover as the opinions evolve in time, the individuals that may interact with a given one also change with time.

The best studied bounded confidence models are the *Deffuant-Weisbuch* (DW) model^12^ and the *Hegselmann-Krause* (HK) model^13^. In both models the opinion of agent $i$ is coded in a continuous variable and each agent may be influenced only by others whose opinion differs from his at most by a quantity called *confidence* ${\varepsilon }_{i}$. Unlike the DW model, which considers pairwise interactions, in the HK model agents are synchronously influenced by all others within their confidence range.

The most studied variant of the latter is the case of homogeneous confidence ${\varepsilon }_{i}=\varepsilon$, where large populations will always converge towards a uniform opinion, if the confidence is above a threshold ${\varepsilon }_{c}\approx 0.2$^13,14^. However, a society is not a homogeneous collection of individuals. Some of them are *open minded* and this property may be modelled by relatively large values of their confidence ${\varepsilon }_{i}$, accordingly the *closed minded* ones will have low values of their confidence ${\varepsilon }_{i}$. Therefore heterogeneity of the confidence of single agents is an obvious and well motivated addition to the model. Until now heterogeneity in the HK model has mainly been studied by introducing multiple sub-populations, each of them formed by a homogeneous set of agents characterized by a specific confidence value^15–18^. Some other works study systems with random confidence, with ${\varepsilon }_{i}$ drawn from some distribution^19–21^, or from different distributions for multiple sub-populations^22^. Those studies are usually performed by the means of multi-agent simulations on small statistical samplings (50–100 samples), where each realization represents also a very small system (typically a few hundred agents). An alternative method, the multiple chain Markov model^15^, has been proposed in order to obtain the properties of the infinite system. However, in this model, opinions are discretized which is known to to cause deviations from the HK model^23^ and the probabilities of each opinion are derived from an initial set of agents. Nevertheless, these works seem to indicate that heterogeneity indeed leads to some surprising non-monotonous effects in the dynamical outcomes. In particular, the existence of consensus in regions where the confidence of the agents is below the critical threshold of the homogeneous case has been reported^17^.

In this article, we present a systematic study of the phase diagram of the HK heterogeneous model in the parameter space given by the possible lower and upper bounds of the confidence values of the agents, $({\varepsilon }_{l},{\varepsilon }_{u})$. The system is neither a fully mixed population, nor a fixed network of contacts. Interestingly, it could be thought of as a non-symmetrical, dynamical interaction network, which may become particularly useful to define a method for building clusters of agents of similar opinions. We carefully sample the parameter space for large systems and we are able to obtain very good statistics for the studied quantities. Furthermore, we study the finite-size effects on the dynamics of the model, which reveal complex details of the consensus landscape that only become apparent at system sizes that are larger than those studied before. A careful study of the dynamical trajectories in the opinion space allows us to explain the re-entrant phase observed in the phase diagram. Finally, we describe the algorithm that allows us to study system sizes and sampling statistics that go well beyond the present state of the art.

## Models and Methods

We study the Hegselmann-Krause model (HK), which describes a compromise dynamic under bounded confidence. Each of $n$ *agents* $i$ is endowed of a dynamical continuous variable ${x}_{i}(t)$ representing *opinion* and a fixed *confidence* ${\varepsilon }_{i}$, modeling the heterogeneous idiosyncrasies of the agents. The *neighbours* of agent $i$ are all agents $j$ whose opinions lie inside the interval $[{x}_{i}-{\varepsilon }_{i},{x}_{i}+{\varepsilon }_{i}]$, i.e.

1 $$I(i,\overrightarrow{x})=\{1\le j\le n||{x}_{i}-{x}_{j}|\le {\varepsilon }_{i}\}\mathrm{}.$$

Note that every agent is a neighbour of itself. In each time step an agent $i$ talks to all its neighbours $j$ and adopts the average opinion of all neighbours, i.e.

2 $${x}_{i}(t+\mathrm{1)}={|I(i,\overrightarrow{x}(t))|}^{-1}\sum _{j\in I(i,\overrightarrow{x}(t))}{x}_{j}(t\mathrm{)}.$$

This update is performed synchronously for all agents, although a sequential random update is possible. The latter typically leads to longer convergence times, as single agents are left behind and may, for small values of $\varepsilon$, persist as isolated clusters in the final state. However, besides these effects, the observations which we describe in the following sections are qualitatively robust against the update schedule.

The dynamics leads to a stable configuration, where the agents settle into one or several *clusters*, (a cluster being a group of agents with the same opinion). The situation in which only one giant cluster exists is called *consensus*, that with two clusters is called *polarization* and with more clusters it is called *fragmentation*. For the homogeneous case it is well known that above a critical ${\varepsilon }_{c}\approx 0.2$ large systems will always converge to consensus and never reach consensus below this threshold^10^.

While the original HK model uses homogeneous confidence ${\varepsilon }_{i}=\varepsilon$, here we draw each ${\varepsilon }_{i}$ from an i.i.d. uniform distribution bounded by two parameters ${\varepsilon }_{l},{\varepsilon }_{u}$ with ${\varepsilon }_{l}\le {\varepsilon }_{u}$. The choice of the lower bound parameter, ${\varepsilon }_{l}\ge 0$, determines how closed minded the most closed minded agents are and the value of the upper bound parameter, ${\varepsilon }_{u}\le 1$, determines how open minded the most open minded agents are.

We are mainly interested in the effects of heterogeneity on consensus for the whole $({\varepsilon }_{l},{\varepsilon }_{u})$ space. To evaluate this, we look at the relative size of the largest cluster $S$ averaged over all simulated realizations, $< \,S\, >$. A value near $1$ indicates consensus, smaller values indicate polarization or fragmentation.

### Tree-based updating scheme

One difficulty in simulating the HK dynamics is that each time step, i.e. the update of all $n$ agents, requires ${\mathcal{O}}({n}^{2})$ operations such that the simulation of large system sizes quickly becomes infeasible. There are attempts to solve this problem with algorithms which are faster, but have some drawbacks, such as not simulating the actual dynamics, but generating an approximation for the $n\to \infty$ case^24^ or imposing a discretization of the opinion variables^23^.

Here, we apply an algorithm which is much faster than the naive approach for typical realizations, while preserving the continuous character down to the precision of the data types used. This enables us to simulate larger sizes with far better statistics than other contemporary studies of the HK model. Its fundamental idea is that to update agent $i$ we do only have to look at the agents within its confidence interval $[{x}_{i}-{\varepsilon }_{i},{x}_{i}+{\varepsilon }_{i}]$, which are typically far fewer than $n$ for small values of ${\varepsilon }_{i}$ – but in the order of ${\mathcal{O}}(n)$ in the worst case.

To achieve this in an efficient way, we maintain a *binary search tree* (BST) [ref.^25^, p.~286] of all opinions ${x}_{j}(t)$. This data structure can be used to find all entries within a range of values efficiently [ref.^26^, p.~412]. Since, in order to update the opinion of agent *i*, we need to find all agents whose opinion is within the range $[{x}_{i}-{\varepsilon }_{i},{x}_{i}+{\varepsilon }_{i}]$, this data structure is exactly what we need.

**Figure 1:**
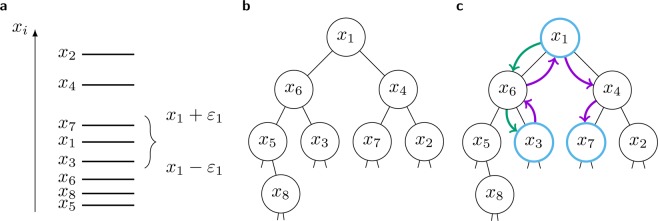
(**a**) Example state of $n=8$ agents, with highlighted confidence interval of agent $i=1$, which includes agents 3 and 7. (**b**) BST obeying the order property in respect to the opinions ${x}_{i}$. (**c**) Sketch of the in-order traversal of the range $[{x}_{1}-{\varepsilon }_{1},{x}_{1}+{\varepsilon }_{1}]$. Green arrows indicate the steps necessary to find the lower bound. Violet arrows show the steps necessary for the traversal. Blue nodes are the ones collected by the algorithm and used to update the opinion of agent $i=1$.

Let us recall the definition of a BST. A *tree* consists of a hierarchical arrangement of *nodes*. Each node is assigned a unique *key* (and possibly additional data). For simplicity, we will refer to a node as *greater* than another node if the key of the first node is greater than the key of the second node and analogously for *smaller*. All nodes, except the *root* (${x}_{1}$ in Fig. 1), have exactly one *parent* node one *level* above and at most two connected nodes called *children* one level below, as sketched in Fig. 1. The children are called *left* and *right* child. The defining property of a BST is the *order property* with respect to the nodes’ keys: in a BST the left child is always smaller than its parent and the right child is always greater than its parent.

For our application, each node represents an agent $i$ and its key is the opinion ${x}_{i}$ of the corresponding agent. An example is sketched in Fig. 1, which should foster intuition about the algorithms explained in the following paragraphs. Note that removal and insertion of nodes into a BST can be done efficiently using standard algorithms^25^.

A BST with $M$ levels can store at most ${2}^{m}-1$ nodes. A tree with $n$ nodes is called *balanced*, if it only has $m={\mathcal{O}}(\mathrm{ln}\,n)$ levels. Since the order property allows us to find any node using only one comparison per level, this allows very efficient search, hence the name. Further, it can also be used to *traverse* all nodes whose keys are within a given range of values, sorted according to their keys. To traverse the range $[l,u]$, one starts at the root. We will call the node where we are at the *current* node $c$. The main idea of the range traversal is to cut whole subtrees from the tree, if all their nodes are smaller than the lower bound $l$ or larger than the upper bound $u$. This can be expressed very compactly with a recursive function:

1. if $c > l$, it calls itself on its left subtree (otherwise all nodes of its left subtree would be smaller than $l$)
2. if its own key is inside the range, it stores its key to be handled later
3. if $c < u$, it calls itself on its right subtree (otherwise all nodes of its right subtree would be larger than $u$)

In other words, this algorithm will use the shortest (and only) path through the tree to find the smallest node larger or equal to the lower bound $l$ and collect every node in ascending order until it encounters a node larger than the upper bound $u$. Consecutively, the $M$ nodes which were collected during the traversal can be used to calculate the new opinion of the agent. During this procedure, we only had to inspect ${\mathcal{O}}(logn+M)$ nodes.

Since BSTs can only store elements with unique keys, we have to handle the case of multiple agents having the same opinion within the precision of the used datatype. Therefore, we save at each node of the tree, additionally to the opinion, a *counter*, which keeps track of the number of agents which have this opinion and are therefore represented by this node. Note that this additional information has no influence on the order of the nodes. Correspondingly, this counter has to be handled as a weight when calculating the new opinion of the active agent.

When the opinion of an agent is updated, the tree must be updated by removing its former opinion (or decreasing its counter by one) and inserting its current opinion (or increasing its counter by one). Both operations can be performed in time ${\mathcal{O}}(\log (n))$, and have to be performed once for each agent in every time step (except ${x}_{i}(t)={x}_{i}(t+\mathrm{1)}$). This algorithm therefore has a typical time complexity for one step between $\Omega (n\,\log (n))$ in the best case, if each agent has at most ${\mathcal{O}}(\log (n))$ neighbours with distinct opinions (i.e. when almost all agents condensed into clusters), and ${\mathcal{O}}({n}^{2})$ in the worst case, i.e. in the beginning when each agent has ${\mathcal{O}}(n)$ neighbours.

Note that the performance of this algorithm benefits from two independent effects. First, for agents with small confidence ${\varepsilon }_{i}$ we profit from the reduced number of opinions we need to look at to determine the average opinion of its neighbours. Second, the HK model tends to form clusters (within the chosen numerical precision) quickly, especially for large ${\varepsilon }_{i}$. Since clusters are represented in the BST as a single node with a high counter, the computational cost to calculate the average opinion is greatly reduced.

A commented minimal implementation and pseudocode for the tree range traversal is provided in supplementary information SI1^1^^1^. There, to further speed up this algorithm, we use a B-tree [ref.^25^, p.~484] instead of a BST.

## Results

We simulated the system for a broad range of system sizes $64\le n\le 131072$ and we carefully explored the parameter space of confidence intervals. For each run, we have drawn the confidence parameter of each agent ${\varepsilon }_{i}$ uniformly from an interval bounded by $({\varepsilon }_{l},\,{\varepsilon }_{u})\in \mathrm{[0,}\,\mathrm{0.35]}\times \mathrm{[0,}\,\mathrm{1]}$. The simulations are run until the opinions, represented with 32 bit IEEE 754 float data-types, converge. The convergence criterion we use here requires that the sum of the changes over all the agents is below a threshold, i.e. ${\sum }_{i=1}^{n}|{x}_{i}(t-\mathrm{1)}-{x}_{i}(t)| < {10}^{-4}$.

Clusters are composed of agents holding the same opinion within a tolerance range of 10^−4^. We have checked that the results are robust when using different clustering criteria, e.g. binning the opinion space, which is briefly shown in supplementary information SI2.

**Figure 2:**
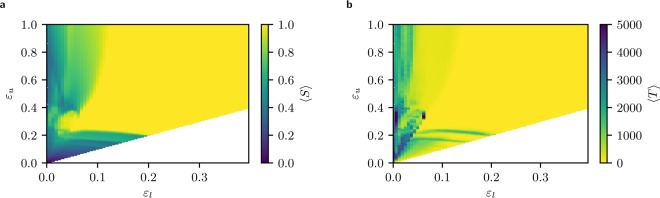
Left: Average relative size of the largest cluster $< \,S\, >$. Right: Average convergence time $< T >$. These data are collected for $8224$ pairs of $({\varepsilon }_{l},{\varepsilon }_{u})$ for a system of $n=16384$ agents and averaged over $1000$ realizations. Note that three parameter pairs at ${\varepsilon }_{l}\,\mathrm{=\; 0}$ did not converge in reasonable computing time for all realizations. To avoid selection bias, they are therefore omitted and marked in white.

Figure 2 shows a heat map of the average size of the largest cluster $< S >$ for each of the $8224$ points that we have simulated in the parameter space. The results shown by the heat map at each point $({\varepsilon }_{l},{\varepsilon }_{u})$ correspond to the average over 1000 simulated samples, which differ in their initial conditions although all represent the same society, where the heterogeneous confidence parameters of the agents ${\varepsilon }_{i}$ have been uniformly drawn from the same interval. Note that the aspect ratio is not unity. While we concentrate on uniformly distributed confidences, supplementary information SI2 shows that qualitatively the same effects occur for other distributions, including heavy tail ones.

The region not shown here ($\mathrm{[0.35,}\,\mathrm{1]}\times \mathrm{[0,}\,\mathrm{1]}$) corresponds to values of confidence intervals that always lead to consensus. This is expected, as both bounds are far above the critical value of the homogeneous case. The white triangle in the lower right corner consists of impossible intervals, where the lower bound would be higher than the upper one. The diagonal elements correspond to the homogeneous case, i.e. ${\varepsilon }_{l}={\varepsilon }_{u}$. One can see that on this diagonal, $< S >$ changes from $1$ to approximately $\mathrm{1/2}$ around ${\varepsilon }_{l}={\varepsilon }_{u}=0.2$, showing the transition from consensus to polarization that has been found in the homogeneous HK model^10^.

The interesting results are situated on the left of the map, where a *re-entrant consensus region* occurs around the point $\mathrm{(0.05,}\,\mathrm{0.3)}$. This reveals a non-monotonous behaviour of the system as the fraction of confident agents increases in the population. Let us examine the map by considering a fixed value of ${\varepsilon }_{l}=0.05$ while varying the upper value ${\varepsilon }_{u}$. We start at the homogeneous case ${\varepsilon }_{u}=0.05$, where we find fragmentation (dark). As ${\varepsilon }_{u}$ increases, a light region is encountered showing strong consensus. This is expected as more agents with an increasing confidence (having a larger number of neighbours) enter the system, contributing to integrate agents with low confidence into the consensus group. However, as ${\varepsilon }_{u}$ increases further, consensus surprisingly disappears (colour gets darker) although the fraction of confident agents and the magnitude of their confidence is even larger.

**Figure 3:**
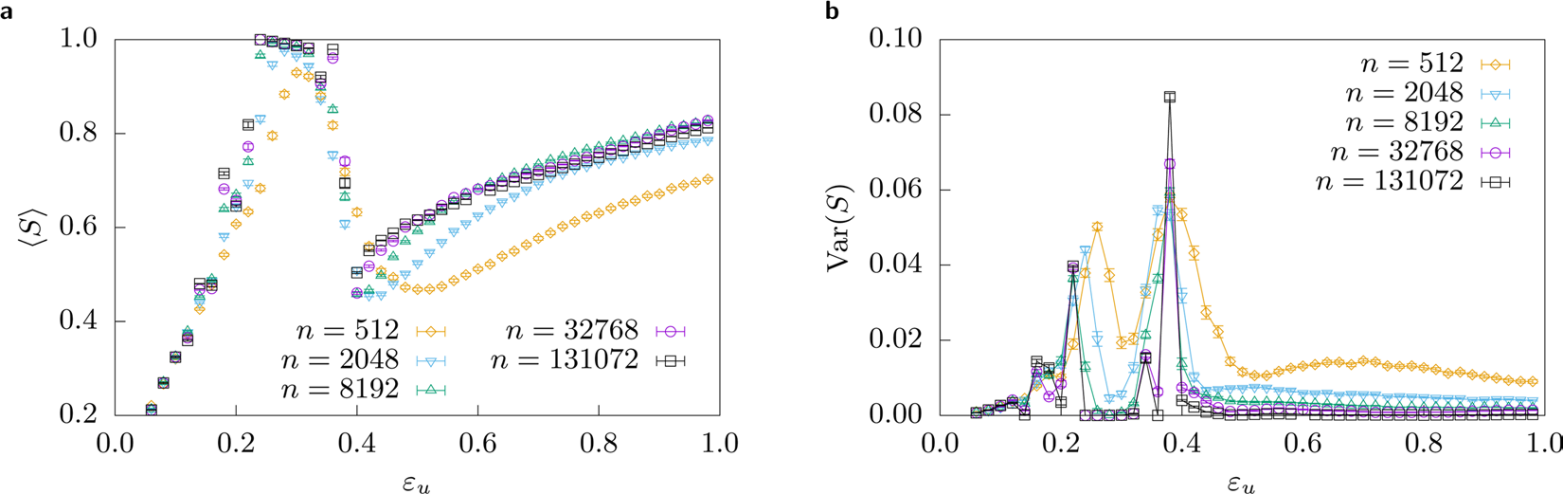
Left: Average size of the largest cluster $< \,S\, >$. Fixed ${\varepsilon }_{l}=0.05$ for varying values of ${\varepsilon }_{u}\in [{\varepsilon }_{l},\,\mathrm{1]}$; this corresponds to a straight vertical line in Fig. 2 through the re-entrant consensus phase. The consensus region is robust with increasing system sizes. Also note that the behaviour is highly complex as the behaviour with increasing system size is not always monotonous, e.g. at ${\varepsilon }_{u}=0.2$ or ${\varepsilon }_{u}=0.42$. Right: Variance of the size of the largest cluster $Var(S)$. The transition points into consensus and out of consensus can be located at the peaks of the variance. Using the finite-size behaviour, we estimate them for ${\varepsilon }_{l}=0.05$ at ${\varepsilon }_{u}=0.22(1)$ and ${\varepsilon }_{u}=0.38(1)$. Lines are just guides to the eye.

The left panel of Fig. 3 shows the evolution of the order parameter $< \,S\, >$ with ${\varepsilon }_{u}$ for a vertical cut in the region of the re-entrant phase of the heat-map shown in Fig. 2 for different system sizes. For a system that contains very closed minded agents, when the fraction of open minded agents increases, the size of the largest cluster also increases leading to consensus, however as the fraction of open minded agents along with their corresponding confidence values increases further, consensus is lost.

An additional small peak, which grows with the system size, is visible on the left of the consensus peak, this reflects the rich structure observed in the phase diagram between ${\varepsilon }_{l}\approx 0.13$ and ${\varepsilon }_{l}\approx 0.18$. Although these fine structures call for more inspection, here we focus on the dominant structure, the re-entrant consensus phase.

The right of Fig. 3 shows the fluctuation of the order parameter $< S >$. There are two visible peaks, which indicate the location of the two transitions, in and out of the re-entrant phase. Interestingly, the finite-size behaviour signals a real double transition as the peaks do not decrease and separate with increasing size, suggesting that this is not just a finite-size effect, but that it remains in the thermodynamic limit, i.e. the $n\to \infty$ limit. Using these data, we estimate the re-entrant phase to span the interval ${\varepsilon }_{u}\in \mathrm{[0.22(1),}\,\mathrm{0.32(1)]}$ for fixed ${\varepsilon }_{l}=0.05$.

**Figure 4:**
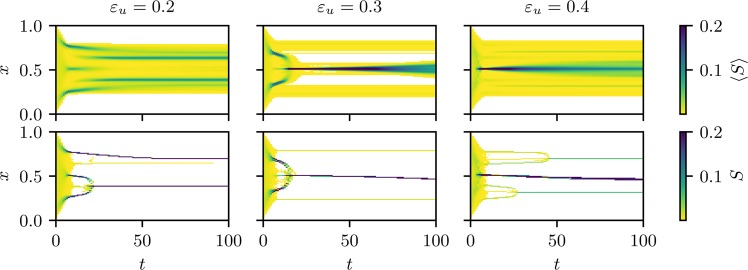
We look for fixed ${\varepsilon }_{l}=0.05$ and $n=16384$ at three values of ${\varepsilon }_{u}\in \mathrm{\{0.2,}\,\mathrm{0.3,}\,\mathrm{0.4\}}$ (from left to right) corresponding to one value left of the peak, one at the peak and one right of the peak of Fig. 3. The images show which fraction of agents have a given opinion $x$ for each time step. Note that the colour scale is truncated at $0.2$ to generate images with a good contrast in the interesting region at small times, such that the darkest colour can also represent values larger than $0.2$. The time axis is also truncated to focus on the most interesting region of small times, i.e. not the whole range until reaching a stationary state is visualized. Top: aggregated statistics over $10000$ realizations of the initial conditions. Bottom: single trajectories.

In order to explain this paradoxical result, a careful examination of the dynamic behaviour of the agents’ opinions is necessary. In Fig. 4 we show the early evolution of opinions for systems corresponding to three different values of ${\varepsilon }_{u}$ in the re-entrant region. The top row corresponds to averages over $10000$ realizations of the initial conditions, while the bottom row illustrates the evolution of opinions with single example realizations.

The mechanism leading to the observed behaviour is rooted in the different characteristic times that open and closed minded agents need to join a majoritarian opinion strand. The latter take more time to reach the consensus opinion as they need to meet agents within their narrow confidence interval. This is at the origin of the bell shaped structures in the bottom left panel of Fig. 4

These structures enable closed minded agents from the whole region over which the bell spans, to join a strand and evolve. If the strand counts enough open minded agents, they can pull it towards a consensus opinion, bringing with them the closed minded agents that have an opinion close to theirs. This is what happens in the bottom middle panel of Fig. 4 at ${\varepsilon }_{u}=0.3$. Also the signature of this structure is clearly visible in the average over many realization in the top middle panel. The two smaller strands visible in this panel contain only 3% of the agents. As it is very frequent that the central cluster converges to one of them, the averaged picture shows an apparent diffusion of the central opinion for long times. Moreover we expect the small clusters to vanish in the thermodynamic limit, since the peak of Fig. 3a shows an upward trend for increasing system sizes.

Note that agent *i* can in each time step move at most ${\varepsilon }_{i}$, such that closed minded agents need to see other agents for at least a few iterations to be able to change their opinion from one extreme to the consensus opinion at $0.5$. More open minded agents (like those that appear when increasing ${\varepsilon }_{u}$) will evolve very quickly to a central opinion, because they can interact with a large fraction of the other agents and therefore are able to jump directly into the centre. As a consequence closed minded agents are left behind and are not able to join the consensus opinion. This is the situation depicted in the right panels of Fig. 4.

**Figure 5:**
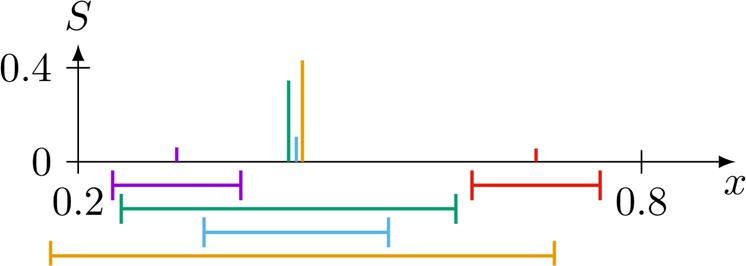
Final state of the single trajectory evolution at ${\varepsilon }_{u}=0.4$ shown in Fig. 4. Each cluster is shown in a different color, the height corresponds to its size $S$ and a typical confidence ${\varepsilon }_{i}$ of a member of that cluster is shown below the plot. The split of the central strand into three clusters is clearly visible (see text for an explanation taking the confidences into account).

The case of ${\varepsilon }_{u}=0.4$, shown on the right of Fig. 4, seems to show that the central strand contains about 90% (the two secondary clusters have a size of $\approx \mathrm{5 \% }$ each), which is an apparent contradiction with the mean cluster size $< S > \approx 0.5$ shown in Fig. 2. The solution to this discrepancy is that the central strand located at an opinion $x\approx 0.44$ splits in the stationary state to three very close clusters, therefore there are 5 clusters in total, which are composed of different groups of agents. This final state is visualized in Fig. 5.

The largest cluster of the central strand contains about $\mathrm{50 \% }$ of the agents (yellow in Fig. 5), mainly having large confidence intervals, able to interact with agents of the small strands located at $x\approx 0.32$ (violet) and $x\approx 0.68$ (red). Another cluster contains about $\mathrm{30 \% }$ of the agents (green), which can interact only with the bottom strand at $x\approx 0.32$ (and all agents of the central strand), but not with the upper one at $x\approx 0.68$, such that it will converge to a slightly lower opinion. The last cluster contains about $\mathrm{10 \% }$ of the agents (blue) which can only interact with those in the central strand (their opinion being in the middle of the other two clusters forming the central strand). Therefore, the size of the largest cluster of this realization is $S\approx 0.5$, the value observed in the valley of Fig. 3. Note that this effect can easily be missed when using a more discrete clustering criterion, like binning with too few bins. Also note that in the ${\varepsilon }_{u}=0.3$ case the secondary clusters, which are already significantly smaller in this example, become smaller in the thermodynamic limit. In contrast, at ${\varepsilon }_{u}=0.4$ the size of secondary clusters stays roughly constant with increasing system size, such that this effect will not vanish in the thermodynamic limit. That means even if we count the central strand as a single cluster, $< S >$ would not grow beyond $< S\, > \approx 0.9$, in contrast to ${\varepsilon }_{u}=0.3$ where it convincingly converges to $< S > \to 1$. A demonstration of this by using a different method to determine clusters, which will always treat the central strand as a single cluster, is shown in the supplementary information SI2 and exhibits the same telltale phase transition signature at the same threshold between ${\varepsilon }_{u}\,\mathrm{=\; 0.3}$ and ${\varepsilon }_{u}\,\mathrm{=\; 0.4}$.

Figure 3b confirms these results: average convergence time in the region of re-entrant consensus is much larger than on neighbouring regions, illustrating the extra time needed by closed minded agents to join the consensus strand *pulled* by open minded agents.

Interestingly, the bell shapes observed in the evolution of opinions, which help to integrate isolated agents into a single strand, are observed for all the parameters shown here. However, when ${\varepsilon }_{u}$ is either too low or too high, these structures are mainly formed by agents with very low confidence, in the first case just because they are the majority of the population and in the second because agents with large confidence have already joined the main strand. For intermediate values of ${\varepsilon }_{u}$, these bell structures contain both open and closed minded agents, and the former may bring the latter into the final consensus strand.

Now, that we understood the main structure of the phase diagram, we can take a more detailed look. There are apparently large horizontal structures visible in Fig. 2 closely below ${\varepsilon }_{u}\approx 0.2$. In vertical slices, these correspond to small local minima and maxima, which can be recognized in Fig. 3 in the left increasing flank. Note that the maxima increase with increasing system size, while the rest of the flank does not, leading to local minima. This is even better visible in the corresponding variance, where small maxima below ${\varepsilon }_{u}=0.2$ arise and persist for larger system sizes. This hints that these structures do not vanish in the thermodynamic limit and are properties inherent to this model. This suggests a very rich behavior that would be worthwhile to study in the future.

In a previous work, where the HK model was studied using an “interacting Markov chain” instead of agent-based simulations^15,27^, an effect called there “consensus strikes back” was reported. This effect consisted in a consensus phase observed in the homogeneous case for a very narrow range of the confidence parameter $0.152\le \varepsilon \le 0.174$.

**Figure 6:**
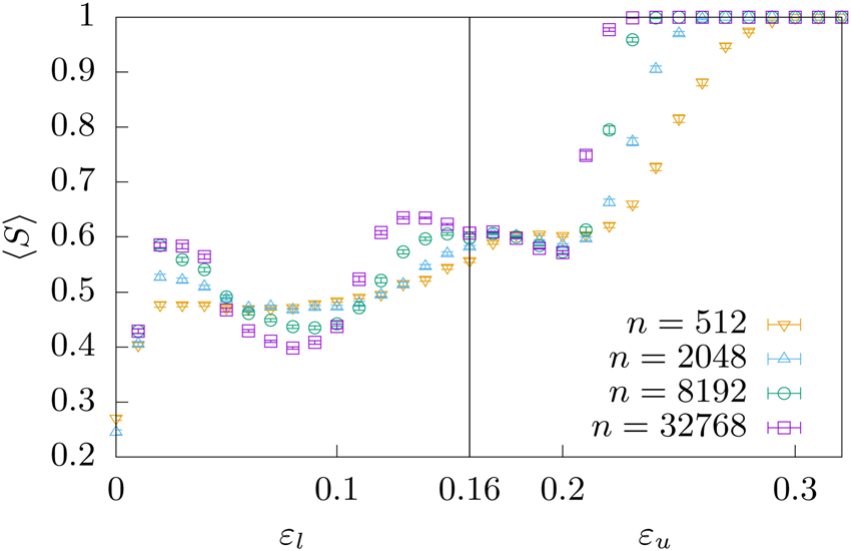
Average size of the largest cluster $< S >$. The left half shows ${\varepsilon }_{u}=0.16$ fixed and ${\varepsilon }_{l}$ varying, the right half shows ${\varepsilon }_{l}=0.16$ fixed and ${\varepsilon }_{u}$ varying. The position marked by the vertical line is the homogeneous case ${\varepsilon }_{l}={\varepsilon }_{u}=0.16$. This corresponds to a straight line in Fig. 2 reflected at the diagonal.

Since one of the aforementioned local maxima appearing in Fig. 3 along with its corresponding fluctuation is located at ${\varepsilon }_{u}\approx 0.16$ for all sizes, we have investigated our model in order to see whether we observe such effect. Figure 6 shows finite size behaviour of $< S >$ for different systems where one bound (either ${\varepsilon }_{l}$ or ${\varepsilon }_{u}$) is fixed to $0.16$ while the other bound is indicated in the horizontal axis. In spite of the fact that, for large systems, there is a slight increase in $< S >$ as one approaches $\varepsilon \approx 0.16$ with the lower or the upper bound of the interval, the value of the largest cluster is far from consensus so as to conclude about the presence of the aforementioned effect.

## Conclusions

We have performed a very detailed characterization of the phase diagram of the heterogeneous Hegselmann-Krause model by means of an efficient algorithm that allows the simulation of large samples. In this way we were able to obtain very good statistics and to investigate finite-size effects overcoming the size limitations of previous works. Our results reveal a non-monotonous behaviour with a re-entrant consensus phase in the region of the parameter space where fragmentation is expected.

Previously, the HK model or closely related models with two different values of the bounded confidence coexisting in the population have been studied, also revealing a non-monotonous behaviour depending on the amount of open and closed minded agents^15,17^. Here, by the means of the simulation of large systems, we characterize the rich behaviour of the fully heterogeneous HK model, and we identify the region $[{\varepsilon }_{l},{\varepsilon }_{u}]$, where the consensus phase clearly enters in the region, in which one would not expect consensus based on the behaviour of the homogeneous case. In particular, we find that increasing the proportion of open minded agents may lead to a loss of consensus, provided closed minded agents are still in the system. We were able to explain this counter-intuitive observation with a careful study of the opinion evolution. Its origin is the slow movement of closed minded agents in the opinion space. When the system contains a large fraction of very open minded agents, who converge very quickly to a majoritarian opinion, the closed minded agents are left behind and full consensus is precluded. On the contrary it is easier to reach consensus when the closed minded agents coexist with a fraction of moderately open minded ones, who converge to the majoritarian opinion much slower. This relative slow convergence allows for the interaction with the closed minded agents who are slowly dragged to the majoritarian opinion.

We have performed several robustness tests that show that our results qualitatively hold for different probability distributions of confidences, including heavy tail ones, and also for different ways of computing the size of the largest opinion cluster. In particular, we observe that although the main mechanism leading to the dramatic loss of consensus we observe is a split-up of a central strand through interaction with small secondary clusters far away, a sharp transition, of a smaller amplitude is still observed when ignoring this split (details can be found in SI2).

We believe that the introduction of quenched disorder by increasing the complexity of the HK model offers a lot of potential for further studies. The high-resolution phase diagram we calculated shows fine structures that we did not explain here, such that open questions remain. An example is the evidence of a tendency towards consensus leading to horizontal structures in the phase diagram at ${\varepsilon }_{u}\approx 0.18$ and ${\varepsilon }_{u}\approx 0.13$. Another one is the “peninsula-like” structure showing that fragmentation appears into the re-entrant consensus region visible in Fig. 2a around $\mathrm{(0.18,0.39)}$, and much more evident in the maximum of convergence time (Fig. 2b). Moreover, variations of the model that introduce a network of social ties, which limits the possible neighbourhoods of any agent, or the integration of cost that the agents must bear for changing their opinion, are work in progress.

## Supplementary information

SI1.

When open mindedness hinders consensus. Supplementary Information 2.

## Footnotes

1 see also https://github.com/surt91/hk_tree
