# Supplementary material for: When open mindedness hinders consensus
Source: Sci Rep. 2020 May 19;10:8273. doi: 10.1038/s41598-020-64691-0 (PMC7237493; doi:10.1038/s41598-020-64691-0)
Supplement: Supplementary file 1 — SI1. [file 41598_2020_64691_MOESM1_ESM.zip › SI1/readme.pdf]

Tree-based Hegselmann-Krause update

The algorithm

For a Hegselmann-Krause update of agent `i` one has to find all agents `j` with an opinion in the range `[x_i - eps_i, x_i + eps_i]`.

The main idea is to maintain a Search Tree of all n agents' opinions. Every time an agent changes its opinion, the tree needs to be updated: Each time the opinion of an agent changes, its previous opinion has to be removed from the tree and its new opinion needs to be inserted such that the order property is preserved. Both can be done in time `O(log(n))`. However, in the tree, one can find efficiently the entry `x_i - eps_i` in `O(log(n))`. As soon as this node is reached, the tree can be traversed in order until a node with a key larger than the upper bound `x_i + eps_i` is encountered.

In a search tree each node has a `key` and at most two children, such that its `left` child's key is always smaller than its own key and the key of its `right` child is always larger than its own key, e.g.:

```
       5
      / \
     /   \
    3     6
   / \     \
  1   4     8
 /         / \
0         7   9
```

For a more complete introduction into tree datastructures and algorithms operating on trees, we highly recommend textbooks on these topics, like *Introduction to algorithms* from Cormen, Leiserson, Rivest, and Stein, or *Algorithms* from Sedgewick, and Wayne.

For our application, each `node` of the tree represents an agent. The `key` of each node contains the value of the opinion of the corresponding agent.

Using a python-esque, recursive pseudocode, this traversal could look like the following. Note that the comparisons in front of the recursive calls cut all subtrees from the traversal, which contain only nodes which are outside of the range, i.e., it uses the order of the tree to find the first element of the range and traverses only until the last. Therefore, this algorithm has (for a balanced tree) a complexity of `O(log(n) + m)`, where `m` is the number of elements in the range. Note that this function does not return a value, but fills the parameter `queue` with the `keys` (opinions) of the encountered `nodes` (agents), which are needed for the update.

```python
def traverse_range(node, queue, lower, upper):
    if not node:
```

```
        return

    # if the current node is larger than our lower bound,
    # search the left subtree recursively
    if node.key > lower:
        traverse_range(node.left, lower, upper)

    # if the current node is within range,
    # store it for later evaluation
    if lower <= node.key <= upper:
        queue.push(node.key)

    # if the current node is smaller than our upper bound,
    # search the right subtree recursively
    if node.key < upper:
        traverse_range(node.right, lower, upper)

# to find the new opinion for a single agent
# with opinion `x` and confidence `eps`
queue = []
traverse_range(root, queue, x-eps, x+eps)
new_opinion = average(queue)
```

Additional to this simple traversal, we have to consider that opinions may occur multiple times, but keys in a search tree need to be unique. This can be solved simply by storing, additionally to the children, also a counter, which keeps track of the number of agents, which hold this opinion. This does, especially after some iterations when many agents settled into clusters of the same opinion, reduce the computation time even more, since fewer nodes have to be traversed.

Since, we have to perform the simulation until all agents reach their final state, this contributes most to the speedup of this algorithm.

Technical details

To ensure that the `finding` phase is fast, the tree needs to be balanced, i.e., the number of nodes on each level should increase exponentially to ensure that finding the lower bound can be done in `O(log n)`. This could be achieved by using AVL- or red-black trees. But generally binary trees are not very efficient for in-order traversal. We decided to use a B-tree instead. Without going into to much detail, a B-tree is a self-balancing generalization of a BST, which stores multiple values inside a node (up to $B+1$, resulting in B children, hence the name). Due to the design of contemporary processors, this leads to a better utilization of caches and is therefore better suited to iterate over contiguous ranges of entries. All used operations still have the same asymptotic complexity as a binary tree.

Example implementation

In the `src` directory there is an example implementation for the tree-based Hegselmann-Krause update. It is written in the rust programming language for its high performance and high-level abstractions. (For installation instructions, see rustup.rs).

This example can be compiled with `cargo build` and the included tests and benchmarks can be executed with `cargo test` and `cargo bench`. When run as a program, it takes some parameters to perform simulations and saves the cluster configuration into a file (more info with `cargo run -- -h`).

The most interesting file is probably `src/hegselmannkrause.rs`, which includes an implementation of the algorithm.
