# Supplementary material for: When open mindedness hinders consensus
Source: Sci Rep. 2020 May 19;10:8273. doi: 10.1038/s41598-020-64691-0 (PMC7237493; doi:10.1038/s41598-020-64691-0)
Supplement: Supplementary file 2 — When open mindedness hinders consensus. Supplementary Information 2. [file 41598_2020_64691_MOESM2_ESM.pdf]

Supplementary Material SI2 of ‘When open mindedness hinders consensus’

Hendrik Schawe[1,*] **and Laura Hernández**[1,+]

[1]Laboratoire de Physique Théorique et Modélisation, UMR-8089 CNRS, CY Cergy Paris Université, France
[*]hendrik.schawe@cyu.fr
[+]laura.hernandez@u-cergy.fr

1 Robustness against different clustering criteria

The order parameter defined in our work, the average size of the largest cluster $\langle S \rangle$, is commonly used as a proxy for the consensus of the system. However, there is not a unique way of determining which agents belong to the same cluster or what consensus is. Here we show that the conclusions of our work are robust face to alternative definitions of consensus.

The results shown for the four phase diagrams of Fig. S1 were obtained for systems of size $n = 16384$ (except (d) with $n = 4096$). In each diagram, each of the 1024 points shown, corresponds to an average over 1000 realizations of the initial conditions.

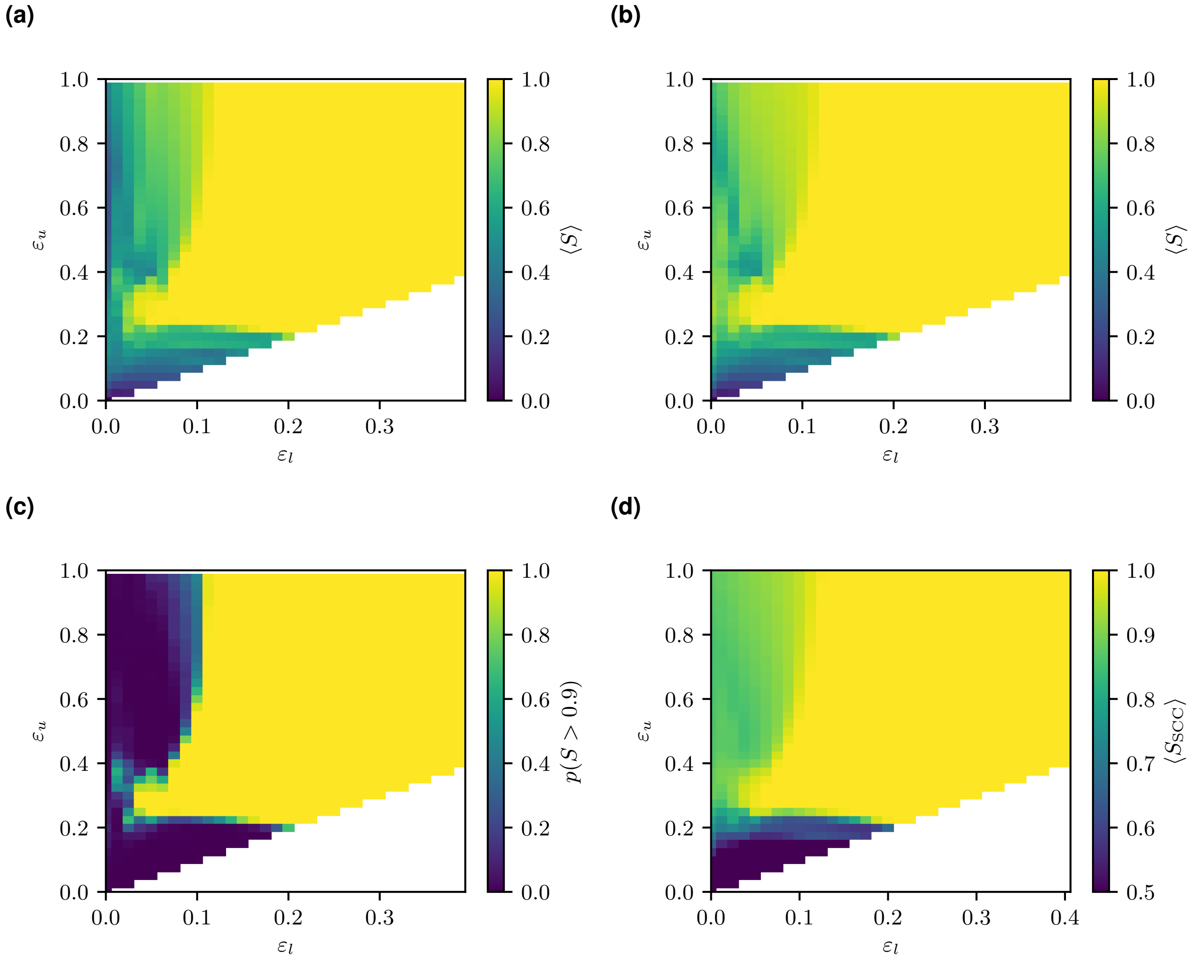

Figure S1. Phase diagrams for 1000 realizations of $n = 16384$ agents with different methods to estimate consensus. (a) Method used in the main text. (b) Opinion is discretized into 100 bins. (c) Consensus is defined as the probability that the largest cluster includes 90% of agents. (d) Consensus is defined by the mutual interaction relation defined in this supplementary material, (see below).

Figure S1(a) shows as a reference the method used in the manuscript: a cluster is defined by a group of agents, which have the same opinion within a tolerance of 10^{-4} .

Figure S1(b) shows an usual method of determining which agents belong to a given cluster, where the opinion space is binned (here in 100 bins) and agents inside the same bin are identified as belonging to the same cluster. Note that this overestimates the size of clusters, e.g., for the example of the main manuscript at $\varepsilon_u = 0.4$, of the three central clusters only two are distinguished in this approach. However, the overall structure is preserved.

Figure S1(c) defines clusters in the same way as we did in the manuscript, but does not show their mean size, but rather the probability that consensus is reached, which is defined – arbitrarily – as the probability that the largest cluster includes more than 90% of all agents. While this, of course, looks different and hides a lot of the structure of the phase diagram, it is suited to demonstrate the extremely robust character of the re-entrant consensus phase.

Figure S1(d) defines clusters as agents which can mutually interact, either directly or indirectly. This is easy to identify as the *strongly connected component* (SCC) of the directed neighbourhood network. The main difference of this definition is that close but split clusters, like the case in the example $(\varepsilon_l, \varepsilon_l,) = (0.05, 0.4)$ of the main manuscript, will be classified as a single cluster. Therefore the size of the largest cluster according to this definition $\langle S_{\text{SCC}} \rangle$ is generally much higher than $\langle S \rangle$ (for this reason, we compressed the color scale). But still the qualitative structure stays the same. To quantify this argument, consider Fig. S2(a), where the sharp phase transition, becoming steeper for larger sizes, appears for $\langle S_{\text{SCC}} \rangle$ at the same position as for $\langle S \rangle$ (Fig. S2(b)) in the main manuscript. This shows that the re-entrant phase is a robust result and not merely the cause of an accidental splitting of the central strand.

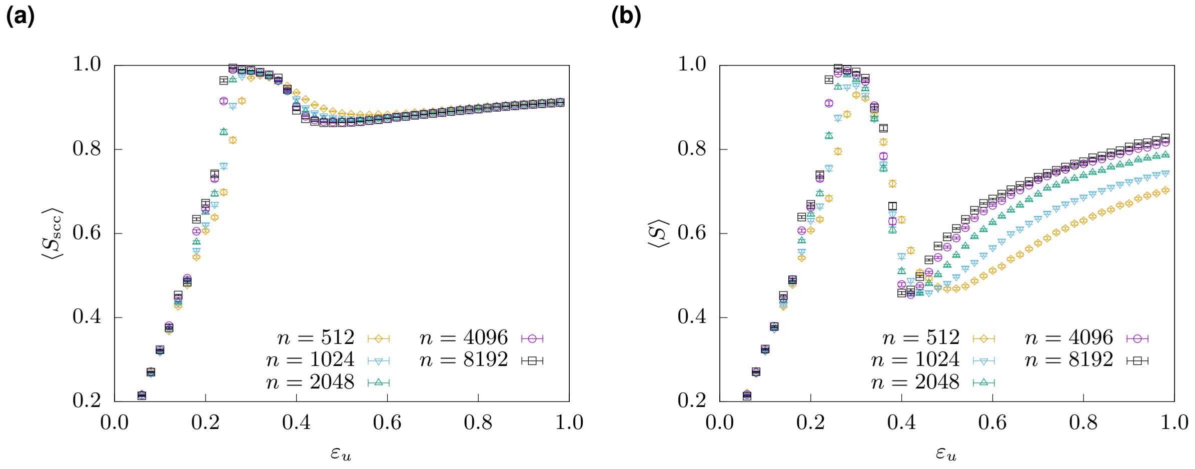

Figure S2. Comparison of the size of the largest cluster at fixed $\varepsilon_l = 0.05$ for two different methods to determine the clusters. (a) Using the mutual interaction criterion SCC. (b) Using the strict definition used in the main manuscript.

2 Non-uniform distributions of confidence ε_i

In the main text we present results obtained for the case where the confidences of the agents, ε_i, are uniformly distributed, a choice imposed for the sake of comparison with previous works. Therefore it is pertinent to test whether our results remain the same when the agents' confidence values are drawn from different probability distributions.

The results shown here correspond to systems of $n = 1024$ agents and 100 realizations of the initial conditions per data point of the phase diagram.

2.1 Gaussian

As the Gaussian distribution, $p(\varepsilon) = \frac{1}{\sigma\sqrt{2\pi}} \exp\left(-\frac{(\varepsilon-\mu)^2}{2\sigma^2}\right)$ is unbounded, it may in principle allow negative confidence values and confidence values larger than 1, which are senseless for our model. Therefore in this test we consider a truncated Gaussian $p(\varepsilon) \propto \exp\left(-\frac{(\varepsilon-\mu)^2}{2\sigma^2}\right) \theta(\varepsilon)\theta(1-\varepsilon)$, with the usual Heaviside step function θ.

Figure S3 shows the phase space parametrized by the mean μ and standard deviation σ. Note that the line at $\sigma = 0$ is the homogeneous case and behaves as expected. Also, a re-entrant consensus region is present when increasing the standard deviation slightly, in particular, we see consensus at a mean confidence μ below the critical threshold.

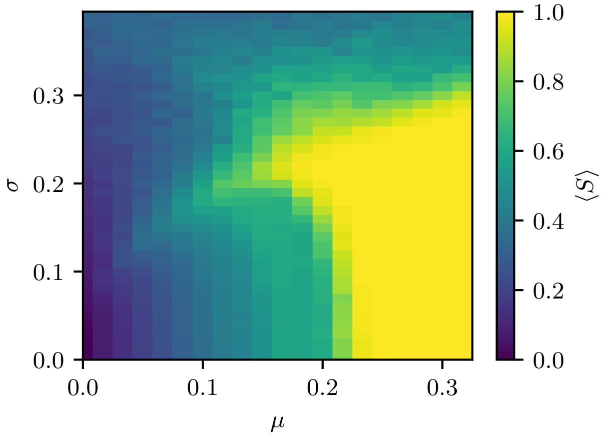

Figure S3. Phase diagram of the HK model with normally distributed confidences.

For very high standard deviations consensus is destroyed again. Like for the the uniform distribution, the larger amount of high confidence agents, which are able to converge to consensus too fast, do not have time to drag less confident agents to consensus with them.

2.2 Pareto distribution

The Pareto distribution $p(\varepsilon) = \frac{\alpha\varepsilon_{\min}^{\alpha}}{\varepsilon^{\alpha+1}} \theta(\varepsilon - \varepsilon_{\min})$ corresponds to a power law density with an exponent of $-(\alpha+1)$. Depending on the value of the exponent it might have extremely heavy tails and therefore it constitutes an alternative to both the uniform and the Gaussian distributions.

The second parameter $\varepsilon_{\min}$ is the lower cut-off somewhat comparable to ε_l of the uniform case. We would expect that we always get consensus for $\varepsilon_{\min} \gtrsim 0.2$. Since the density function will become more concentrated around $\varepsilon_{\min}$ for large values of the exponent, α, it tends to the homogeneous case.

In Fig. S4(a) we observe a loss of consensus for extremely heavy tailed shapes for $\alpha + 1 < 2$. This situation is explained in the same way as in the case of the uniform distribution with high upper bound of confidence ε_u: Many open minded agents are present who can quickly jump to the consensus opinion, leaving the closed minded behind. Note that this correspondence is the reason for the inverted vertical axis. The comparison among the fractions of open minded agents corresponding to the different values of alpha is illustrated in Fig. S4(b). At intermediate $\alpha + 1$ between roughly 2 and 3, where the distribution has fixed average but diverging variance, we always get consensus. Since this is the case of a mixture of closed minded agents and few moderately open minded agents (cf. the lighter tail in Fig. S4(b) compared to the $\alpha + 1 < 2$ case), this supports the conclusion reached in the manuscript. Due to the heavy tail there are always enough open minded agents to pull the slowly evolving closed minded agents into consensus but not enough so as to drive the system to the situation where the open minded agents evolve too fast. For large values of α, where the variance is finite, we have basically a population consisting almost exclusively of

closed minded agents, (cf. the values close to zero in the majority of Fig. S4(b)), leading naturally to fragmentation. This case is analogous to a small value of the upper bound ε_u in the uniform distribution of confidence values. All together, these results qualitatively support the interpretation given in the main text: on one hand, too few open minded agents are not enough to create consensus and on the other, too many open minded agents hinder consensus.

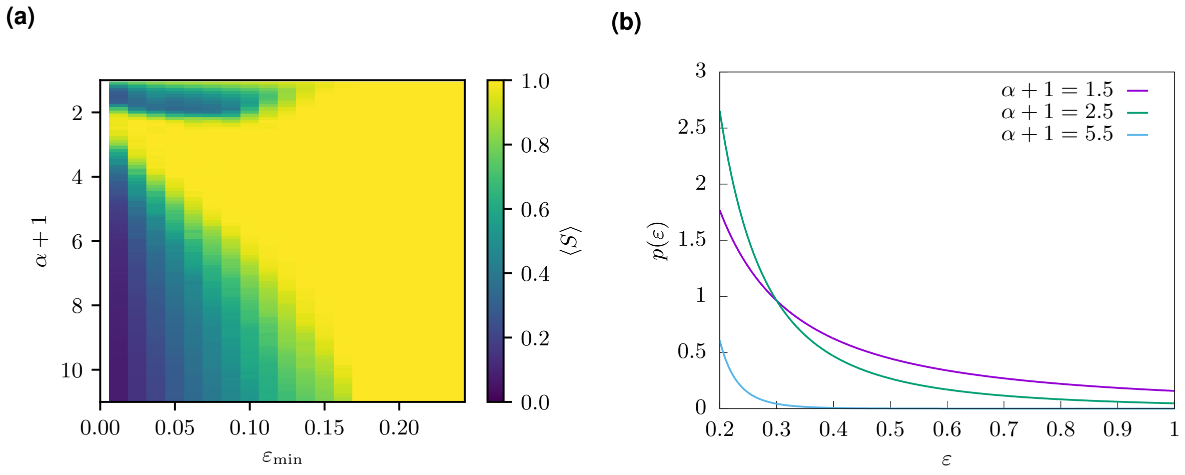

Figure S4. (a) Phase diagram of the HK model with Pareto distributed confidences. (b) Example comparison of the distribution of confidences in the Pareto with different exponents $\alpha+1$. $\varepsilon_{\text{min}} = 0.1$ for all cases. Note that the image concentrates on the tail region for $\varepsilon \leq 0.2$, which corresponds to open minded agents and does not show the peaks of the distributions.

2.3 Bounded power law

Finally, we look at power law distributions with exponent $\gamma = 2.5$, in the parameter range where the distribution has a divergent variance. Therefore we choose the ε_i in intervals bounded by ε_{min} and ε_{max}, i.e., a probability density of $p(\varepsilon) = c\varepsilon^{-\gamma}\theta(\varepsilon-\varepsilon_{\text{min}})\theta(\varepsilon_{\text{max}}-\varepsilon)$ with the normalization constant $c = \frac{-\gamma+1}{\varepsilon_{\text{max}}^{-\gamma+1}-\varepsilon_{\text{min}}^{-\gamma+1}}$. The parameters ε_{min} and ε_{max} are comparable to ε_l and ε_u from the case of the uniform distribution, with the important difference that the probability to choose closed minded agents is here always higher, making them dominant in the population. To make this relation more clear, examples for both distributions are illustrated in Fig. S5(a).

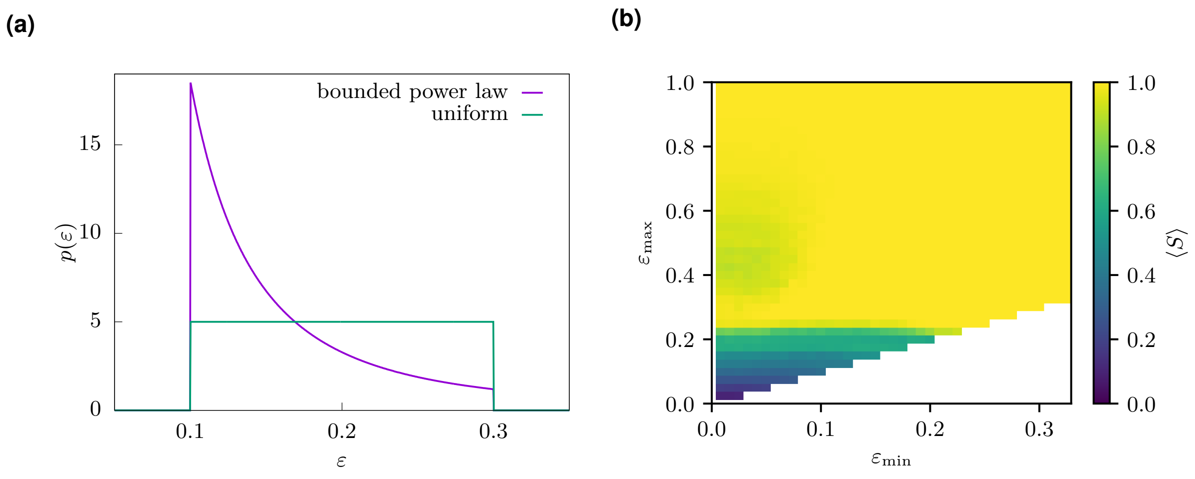

Figure S5. (a) Example comparison of the distribution of confidences in the uniform and bounded power law case. The parameters for both distributions are $\varepsilon_l = \varepsilon_{\text{min}} = 0.1$ and $\varepsilon_u = \varepsilon_{\text{max}} = 0.3$. (b) Phase diagram of the HK model with bounded power law distributed confidences. The negative exponent of the power law here is $\gamma = 2.5$

In Fig. S5(b) we see that consensus is far more prevalent here. For low ε_{min}, increasing ε_{max} integrates open minded agents. But since the probability density of the distribution falls fast for larger confidences ε (cf. Fig. S5(a)) there will always be

only few open minded agents and even fewer very open minded agents, therefore the situation is comparable to the uniform distribution depicted in Fig. 3 of the main text.
